# Shake-it-off: a simple ultrasonic cryo-EM specimen-preparation device

**DOI:** 10.1107/S2059798319014372

**Published:** 2019-11-22

**Authors:** John L. Rubinstein, Hui Guo, Zev A. Ripstein, Ali Haydaroglu, Aaron Au, Christopher M. Yip, Justin M. Di Trani, Samir Benlekbir, Timothy Kwok

**Affiliations:** a The Hospital for Sick Children Research Institute, Toronto, Ontario, Canada; b Department of Medical Biophysics, The University of Toronto, Toronto, Ontario, Canada; c Department of Biochemistry, The University of Toronto, Toronto, Ontario, Canada; d Engineering Science Division, The University of Toronto, Toronto, Ontario, Canada; e Institute of Biomaterials and Biomedical Engineering, The University of Toronto, Toronto, Ontario, Canada

**Keywords:** cryo-EM, specimen preparation, ultrasonic, self-wicking grids, Raspberry Pi, 3D printing, CNC milling

## Abstract

Although microscopes and image-analysis software for electron cryomicroscopy (cryo-EM) have improved dramatically in recent years, specimen-preparation methods have lagged behind. Most strategies still rely on blotting microscope grids with paper to produce a thin film of solution suitable for vitrification. This approach loses more than 99.9% of the applied sample and requires several seconds, leading to problematic air–water interface interactions for macromolecules in the resulting thin film of solution and complicating time-resolved studies. Recently developed self-wicking EM grids allow the use of small volumes of sample, with nanowires on the grid bars removing excess solution to produce a thin film within tens of milliseconds from sample application to freezing. Here, a simple cryo-EM specimen-preparation device that uses components from an ultrasonic humidifier to transfer protein solution onto a self-wicking EM grid is presented. The device is controlled by a Raspberry Pi single-board computer and all components are either widely available or can be manufactured by online services, allowing the device to be constructed in laboratories that specialize in cryo-EM rather than instrument design. The simple open-source design permits the straightforward customization of the instrument for specialized experiments.

## Abstract 2

A method is presented for high-speed low-volume cryo-EM specimen preparation with a device constructed from readily available components.

## 1. Introduction   

During the 1980s, Jacques Dubochet and colleagues established that thin films of protein solutions can be vitrified and imaged by electron microscopy (Dubochet *et al.*, 1988 ▸). Dubochet’s method, which is still standard for cryo-EM, requires a few microlitres of protein solution to be applied onto an EM support grid coated with a carbon or gold film that contains numerous micrometre-scale holes. The majority of solution is then blotted away with filter paper, leaving an ∼500–1000 Å thick aqueous film that is rapidly frozen by plunging into a bath of cryogen. Despite its enormous success, the method has several drawbacks. Firstly, the process is remarkably wasteful. For example, with a 1 MDa protein complex that requires ∼3 ml of sample at ∼3 mg ml^−1^ for an optimal density of particles in images (Rubinstein *et al.*, 2003 ▸), the applied solution contains ∼9 × 10^−6^ g of protein consisting of ∼5 × 10^12^ molecules. If blotted to form a 1000 Å thick film that covers the 3 mm diameter grid, only ∼2 × 10^−9^ g of protein or ∼1 × 10^9^ molecules remain, representing a loss of 99.98% of the material used in the experiment. At present, a typical analysis may include ∼10^6^ molecules, which corresponds to ∼0.00002% of the sample applied. Secondly, the process of blotting specimens with filter paper typically takes several seconds. During this time, the surface area to volume ratio of the specimen increases dramatically, allowing protein interactions with the air–water interface that lead to preferred orientations or denaturation of the sample (Glaeser *et al.*, 2016 ▸; D’Imprima *et al.*, 2019 ▸; Noble, Dandey *et al.*, 2018 ▸; Noble, Wei *et al.*, 2018 ▸). The slow speed of the blotting process also complicates the design of experiments intended for time-resolved visualization of specimens.

Several recent approaches have been described to circumvent some or all of the limitations of the blotting method for specimen preparation. Time-resolved EM experiments have been performed with pressure-driven microfluidic (Feng *et al.*, 2017 ▸; Kaledhonkar *et al.*, 2018 ▸) and voltage-assisted (Kontziampasis *et al.*, 2019 ▸) spraying devices. Sample loss has been addressed by using microcapillaries to apply small volumes of specimen solutions, with the cryoWriter device capable of applying less than 5 nl of sample to a conventional EM grid (Kemmerling *et al.*, 2013 ▸; Arnold *et al.*, 2016 ▸, 2017 ▸; Schmidli *et al.*, 2019 ▸). This method has allowed the impressive determination of a structure of the human 20S proteasome to ∼3.5 Å resolution after isolation from 1 µl of cultured human cells (Schmidli *et al.*, 2019 ▸). Another recently described device applies a specimen to a cryo-EM grid using a purpose-constructed piezoelectric transducer designed to create an aerosol from the specimen by the production of a standing wave (Ashtiani *et al.*, 2018 ▸). A highly developed approach that reduces both the issues of inefficient specimen usage and the long delays between specimen application and freezing was developed at the NIH National Resource for Automated Molecular Microscopy (Jain *et al.*, 2012 ▸; Dandey *et al.*, 2018 ▸). The Spotiton device uses a piezoelectric transducer coupled to a microcapillary, similar to an inkjet printer, to apply a stream of droplets of tens of picolitres onto an EM grid. A recent breakthrough by the same group that dramatically improves the quality of the resulting ice films was the development of ‘self-wicking’ EM grids (Wei *et al.*, 2018 ▸; Razinkov *et al.*, 2016 ▸). These grids are produced by treating copper–rhodium EM grids with an ammonium persulfate solution at basic pH, which leads to the growth of Cu(OH)_2_ nanowires on the copper surface of the grid. The nanowires function to remove excess liquid from the grid, similar to blotting paper, but on a smaller scale and faster. Unfortunately, the process of growing nanowires damages commercially available specimen grids that are already coated with holey carbon or gold films. However, plastic films can be readily produced with regular arrays of holes using a microcontact printing approach (Chester *et al.*, 2007 ▸; Marr *et al.*, 2014 ▸). These films can be deposited on the self-wicking grids and subsequently coated with either carbon or gold (Meyerson *et al.*, 2014 ▸; Russo & Passmore, 2014 ▸). The Spotiton device allows rapid specimen preparation, dramatically reduces the amount of specimen required, and in principle allows time-resolved experiments.

Several aspects of the approaches described above require the precise engineering of mechanical and optical components, complicating construction in laboratories that do not specialize in instrument design. Here, we present a simple and inexpensive cryo-EM specimen-preparation device where samples are applied to self-wicking EM grids as droplets generated from components adapted from a readily available ultrasonic humidifier. The device has several advantages, including rapid specimen preparation, low sample consumption, and the capability to perform time-resolved experiments. Most importantly, the device can be easily adapted for the design of application-specific experiments. In order to facilitate this role, we built the device from standard components and control it with an inexpensive Raspberry Pi single-board computer running a Python program. The designs for all of the custom parts are available as open-source files and the parts can be manufactured, even by a laboratory without access to a technical workshop, using online 3D printing, CNC milling, and printed circuit-board fabrication services. This design philosophy will allow other research groups to adapt the device for other applications. Owing to the use of a piezoelectric transducer to produce droplets of sample, and in homage to the Spotiton instrument that pioneered the use of self-wicking grids, we named our device Shake-it-off or SIO.

## 2. Methods and results   

### 2.1. Overview and process control   

**Figure 1:**
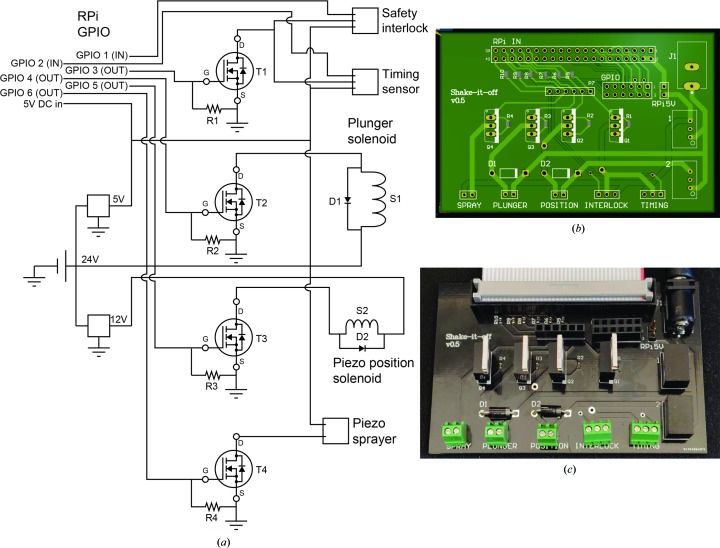
(*a*) Schematic diagram of the SIO control circuit. (*b*) Design for fabrication of the custom-printed circuit board. (*c*) Custom-fabricated printed circuit board.

The SIO device uses four main components to prepare specimens: a piezoelectric sprayer, self-wicking grids, a plunging solenoid with detachable tweezers, and a cryogen bath. The timing of the different electronic processes is controlled by a Raspberry Pi (RPi) single-board computer (Raspberry Pi 3B+; https://www.raspberrypi.org/) running the default Raspbian operating system (v.8, ‘Jessie’). The Python 3 software on the RPi can set the voltage of the general-purpose input–output (GPIO) pins of the RPi to either ‘high’ (3.3 V) or ‘low’ (0 V). A high signal from the GPIO pin applied to the gate of an N-channel enhancement mode power MOSFET (metal oxide semiconductor field-effect transistor) allows the transistor to be used as a high-speed switch, with multiple transistors providing power to the different components of the device when needed (Fig. 1 ▸ *a*). A printed circuit board (Fig. 1 ▸ *b*) was designed with *Altium Designer* v.18 and manufactured by PCBWay (https://www.pcbway.com/). A parts list summarizing all of the components is provided as supporting information.

### 2.2. Ultrasonic dispensing of solutions   

**Figure 2:**
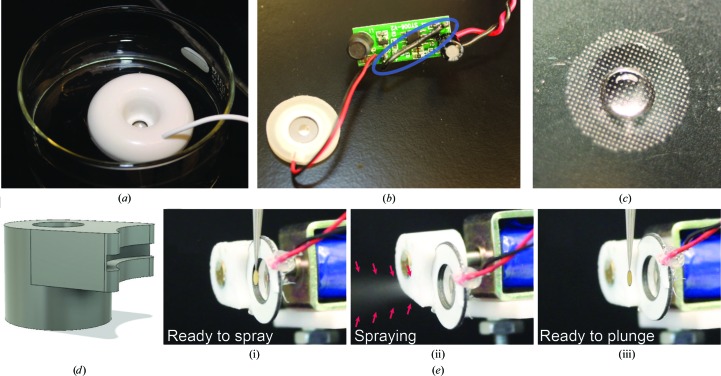
(*a*) USB ultrasonic humidifier containing a piezoelectric transducer and high-frequency generating circuit used to spray specimen. (*b*) The piezoelectric transducer and high-frequency generating circuit extracted from the humidifier shown in (*a*). A wire that allows the circuit to be activated out of water is circled in blue. (*c*) A droplet (1 µl) applied to the piezoelectric transducer surface opposite to the direction of liquid spray. (*d*) Design for the piezoelectric transducer mounting connector. (*e*) Piezoelectric transducer, tweezers and grid in the specimen-application position (i), showing spray from the piezoelectric transducer (ii) and with the piezoelectric transducer retracted in the ready-to-plunge position (iii).

SIO sprays a stream of small droplets onto a self-wicking EM grid with the simple and inexpensive piezoelectric transducer and high-frequency generating circuitry of a commercially available novelty ultrasonic humidifier (Fig. 2 ▸ *a*). The humidifier was designed to be powered from the 5 V USB port of a computer. When placed in water, the electrical conductivity of the water connects an electrode in the device to ground, allowing the device to activate and avoiding damage from energizing the piezoelectric transducer in air for extended periods. To override this safeguard in the humidifier circuit, a wire was added to ground the electrode (Fig. 2 ▸ *b*, circled in blue) so that the circuit can be activated by connection to a 5 V power supply. The piezoelectric transducer of the humidifier has a central array, approximately 3.5 mm in diameter, with numerous small holes that allow liquid to pass through the transducer (Fig. 2 ▸ *c*). In order to spray a grid, the piezoelectric transducer is held in close proximity to the grid, and protein sample (∼1 µl) is applied to the centre of the surface of the transducer facing away from the EM grid. When the high-frequency generating circuit is activated, a small stream of droplets is ejected from the piezoelectric transducer toward the grid. The signal-generating circuit for the piezoelectric transducer used in the SIO device was observed to generate a crude 108 kHz signal, with a peak amplitude of ∼175 V and a duty cycle of ∼11%. The droplet size from this specific piezoelectric transducer has not been characterized experimentally. However, an analysis of related devices showed the production of droplets that decreased from 100 to 10 nm in diameter as the frequency was increased from 200 to 2400 kHz (Kudo *et al.*, 2017 ▸), as well as the production of a smaller population of larger droplets 1–10 µm in diameter (Kudo *et al.*, 2017 ▸; Rodes *et al.*, 2007 ▸). With these higher frequency devices the precise size of the droplets also varied with salt concentration (Kudo *et al.*, 2017 ▸). We found that a 2 µl drop was fully aerosolized after ∼40 pulses of 5 ms, suggesting that each 5 ms pulse dispenses on average ∼50 nl. However, the aerosolization process may not be linear and the volume dispensed may vary with the amount of liquid remaining on the transducer. To prepare grids, we applied ∼1 µl of sample onto the transducer, although it may be possible to use smaller volumes. In principle, ∼1 µl of applied sample can be used to prepare numerous specimen grids. However, it is also possible that some samples could be damaged by multiple exposures to the high-frequency vibrations that they experience from contact with the piezoelectric transducer. While unused samples could be recovered from the transducer and used for other purposes, we usually removed leftover sample with tissue paper following the preparation of each grid. After the removal of sample, the piezoelectric transducer was cleaned further by applying a larger drop of water (*e.g.* 2–3 µl) and energizing the transducer for ∼200 ms five times in a row.

### 2.3. Self-wicking grids   

Self-wicking grids were prepared as described previously by incubating 400 mesh copper–rhodium grids (Maxtaform, Electron Microscopy Sciences) in nanowire growth solution consisting of 750 m*M* sodium hydroxide and 65 m*M* ammonium persulfate for 5 min followed by washing with water (Razinkov *et al.*, 2016 ▸; Wei *et al.*, 2018 ▸). A continuous formvar film with ∼2 µm holes spaced 4 µm centre to centre was prepared and overlaid on the self-wicking grids as described previously (Marr *et al.*, 2014 ▸) and coated with ∼35 nm of gold using a Leica EM ACE200 sputter coater before dissolving the plastic film with chloroform. It may be that during spraying of the grid droplets smaller than the hole size in the gold film pass through the holes, while the larger droplets cover the holes. However, the high density of smaller droplets may also contribute to the formation of a suitable film for cryo-EM once the holes have been blocked by larger droplets.

### 2.4. Grid plunging   

Reproducibly hitting the EM grid with the stream of droplets proved challenging when the grid was held sufficiently far from the piezoelectric transducer to allow plunging of the tweezers. Therefore, the piezoelectric transducer was mounted on a small ‘pull-type’ solenoid. The mount attaching the piezoelectric transducer to the solenoid was designed with *Autodesk Fusion 360* (Fig. 2 ▸ *d*). All 3D-printed components were printed in polycarbonate with a Fortus 450mc 3D printer at the University of Toronto Medstore 3D-printing facility (https://www.uoftmedprint.com/). The solenoid is positioned so that in the spraying position the piezoelectric transducer touches the tweezers and is ∼1 mm away from the grid (Fig. 2 ▸ *e*, i), allowing the spray from the piezoelectric transducer to hit the grid (Fig. 2 ▸ *e*, ii). Removing power from the solenoid allows the spring-driven retraction of the piezoelectric transducer from the grid (Fig. 2 ▸ *e*, iii), providing the necessary clearance for the grid to be plunged into cryogen without the tweezers hitting the transducer. Removing current from an inductive load such as a solenoid can produce a sudden voltage spike across the load. In order to avoid damage to the MOSFET from this voltage spike, a fly-back diode was included in the circuit with a polarity opposite to the applied voltage (Fig. 1 ▸ *a*, diodes D1 and D2).

**Figure 3:**
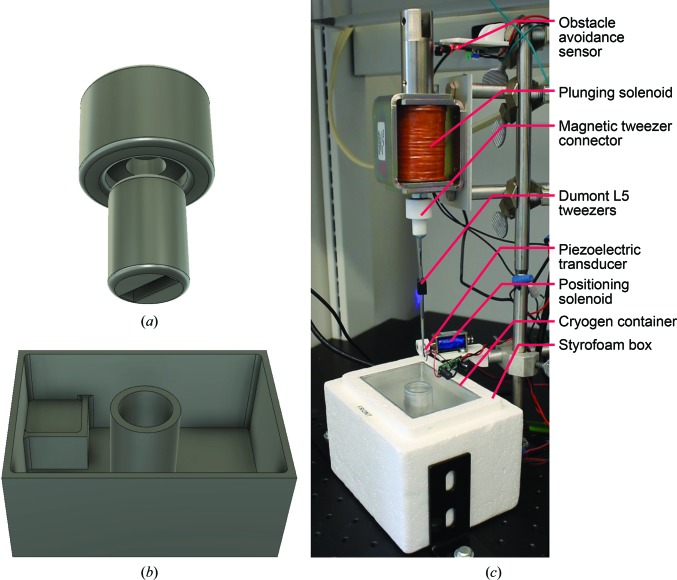
(*a*) Design for the magnetic tweezer connector. The two parts are held together by embedded magnets. (*b*) The 3D design for the cryogen container, which fits within a standard Abcam Styrofoam box, milled from aluminium. (*c*) Fully assembled SIO specimen-preparation device.

Plunging of the grid is performed by a large solenoid with a 30 mm travel distance, identical to the solenoid previously used for the same purpose in the cryoWriter device (Arnold *et al.*, 2016 ▸, 2017 ▸; Kemmerling *et al.*, 2013 ▸). A fly-back diode was also employed for this solenoid. In order to allow the fast and convenient connection and disconnection of tweezers from the plunging solenoid, a magnetic connector was printed (Fig. 3 ▸ *a*). One half of this connector attaches to the plunging solenoid by a nut held in place with epoxy putty, while the other half is attached to the tweezers. The two halves are held together by cylindrical neodymium magnets (6 mm in diameter and 3 mm thickness) secured with epoxy.

### 2.5. Cryogen container   

Freezing of specimens for cryo-EM is typically performed by rapid plunging into liquid ethane cooled by liquid nitrogen. Ethane freezes at liquid-nitrogen temperature and consequently the cryogen containers for specimen-preparation devices need to be designed to keep the ethane in its liquid state. One method for keeping ethane liquid is the immersion of a heating element, as performed in many devices (Bellare *et al.*, 1988 ▸). Alternatively, the cryogen container can be carefully designed so that the thermal contact between the ethane chamber and the liquid-nitrogen chamber is sufficient to keep the ethane liquid but not sufficient to freeze the ethane, as performed with the popular Vitrobot grid-freezing device. This approach removes the need for an immersed heating element but prevents the cryogen container from being manufactured from materials with a high thermal conductivity such as aluminium. Tivol *et al.* (2008 ▸) presented an ingenious solution to this problem: mixing propane with ethane in a ∼60:40 ratio depresses the freezing point of both alkanes so that the mixture does not freeze even at liquid-nitrogen temperature. We designed a liquid-nitrogen reservoir with a propane/ethane bath and manufactured it from a single block of 6061 alloy aluminium by computer numerical control (CNC) milling (https://www.3dhubs.com/). The cryogen container (Fig. 3 ▸ *b*) was designed so that it could be insulated by a small Styrofoam box used as a standard shipping container by the company Abcam. These containers are easily found in many biological research laboratories. In the complete SIO device, the cryogen container is held in place relative to the piezoelectric transducer and plunging solenoid by corner braces on an optical breadboard (Thorlabs) using a vertical post and standard retort-stand boss heads (Fig. 3 ▸ *c*).

Accidentally plunging the sharp tweezers before the cryogen container is in place presents a safety hazard. Therefore, we added a safety interlock to the design. A neodymium magnet was embedded in the Styrofoam of the cryogen container and a miniature reed switch attached to the positioning corner brace for the container. The magnet is held near the switch only when the container is positioned appropriately. The field from the magnet closes the switch, which is detected by the RPi. The software only allows the plunger solenoid to be activated if the cryogen container is in place. There is no shield to protect the experimenter from splashing of the cryogen that may occur during grid plunging. Therefore, safety glasses should always be worn when operating this device.

## 3. Structure determination   

Conditions were identified in which ice suitable for cryo-EM structure determination could be produced consistently using horse spleen apoferritin (Sigma). The full specimen-preparation process is shown in Supplementary Video S1. The concentration of glycerol in the sample was reduced from 50% by diluting 20× in TBS (50 m*M* Tris–HCl, 150 m*M* NaCl pH 7.4) and concentrating 20× with a centrifugal concentrating device with a 100 kDa molecular-weight cutoff membrane. Immediately before use, grids were glow-discharged in air for 2 min at 40 mbar with 25 mA current in a Pelco easiGlow glow-discharge device, with the nanowire surface of the grid facing the plasma of the discharge device. A 1 µl droplet of apo­ferritin solution at 5 mg ml^−1^ in TBS was placed on the back of the piezoelectric transducer. 5 mg ml^−1^ is a typical concentration for apoferritin when used in our laboratory for preparing specimen grids with a conventional grid-preparation device. To apply the specimen to the grid, the high-frequency generating circuit was energized for 5 ms. At 5 ms the piezo was retracted from the grid by de-energizing the piezo-positioning solenoid and the plunging solenoid was energized. An infrared obstacle-avoidance sensor (Keyestudio) mounted on the plunging solenoid, as well as recording a slow-motion video at 2.5 ms per frame with a mobile-phone video camera (OnePlus 6), both suggested a total time from the beginning of spraying to complete immersion of the grid in cryogen of ∼90 ms (Supplementary Video S2). Freezing times of approximately this length with the Spotiton device have been shown to reduce preferred orientations from proteins interacting with air–water interfaces (Noble, Wei *et al.*, 2018 ▸), but we have not demonstrated this advantage here.

**Figure 4:**
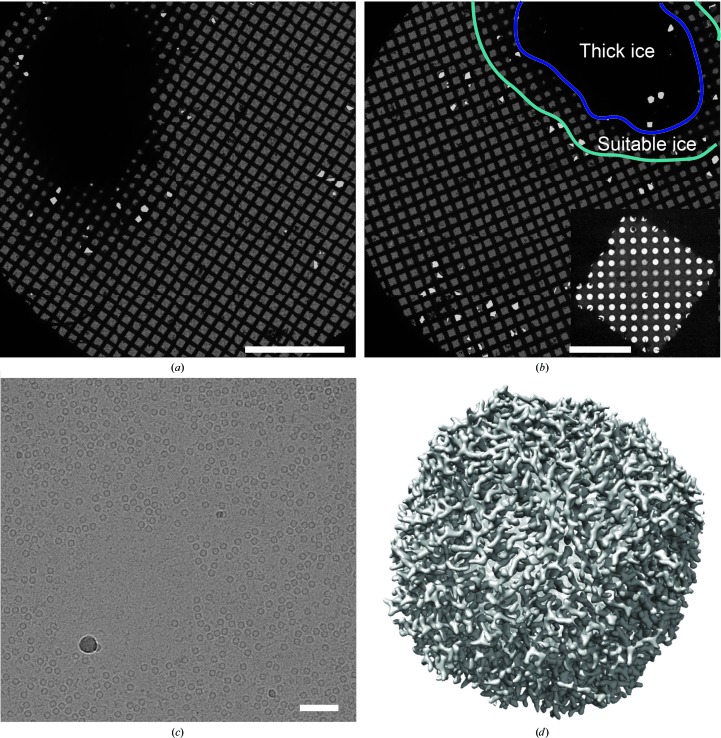
(*a*, *b*) Grid atlases from the Titan Krios microscope showing a large area of thick ice [circled in purple in (*b*)] with a peripheral area of ice suitable for data collection [circled in cyan in (*b*)]. Scale bar, 500 mm. Inset, a grid square from a region with suitable ice for imaging showing some overwicking at its periphery. Scale bar, 20 µm. (*c*) An image from the TF20 microscope showing equine apoferritin particles in ice. Scale bar, 500 Å. (*d*) A 3D map of equine apoferritin at 2.6 Å resolution from the Titan Krios microscope.

Grids were characterized with an FEI Tecnai F20 microscope operating at 200 kV and micrographs were recorded as 15 s movies of 30 frames at 5 electrons per pixel per second and a calibrated pixel size of 1.45 Å per pixel. A data set of 324 high-resolution movies was collected on a Titan Krios G3 electron microscope operating at 300 kV and recorded as 180 frames over 30 s on a Falcon 3EC with an exposure rate of 0.8 electrons per pixel per second, a calibrated pixel size of 1.06 Å per pixel, and a total exposure of 21 electrons Å^−2^. Atlas images of grids (Figs. 4 ▸ *a* and 4 ▸ *b*) show that the device creates an area of thick ice surrounded by a band of grid squares that have ice suitable for data collection. This appearance for grids was typical and did not appear to depend on the sample sprayed. It presumably occurs because the central and most intense region of spray from the piezoelectric transducer deposits more liquid onto the grid than can be removed by the wicking action of the nanowires. It may be possible to reduce the thickness of the ice in this region of the grid by reducing further the time during which the piezoelectric transducer is energized during spraying. An image of a grid square (Fig. 4 ▸ *b*, inset) shows that the edges of the grid square can have holes without ice, possibly owing to excess material being removed by the self-wicking grids, while the centre of the grid square has ice suitable for imaging. Apoferritin particles can be seen in images from holes in this region, which also show some contaminating or damaged particles, probably from the long-term storage of the commercially available apoferritin (Fig. 4 ▸ *c*). Image analysis with *cryoSPARC* v.2 (Punjani *et al.*, 2017 ▸) allowed the selection of 308 752 particle images that were reduced to a data set of 172 469 particle images by 2D and 3D classification. These images were used to calculate a 3D map at 2.6 Å resolution (Fig. 4 ▸ *d*).

## 4. Discussion   

Here, we have shown that a simple device combining 3D-printed and CNC-milled parts, a printed circuit board, and inexpensive consumer components is capable of producing specimens suitable for high-resolution cryo-EM analysis with ∼100 ms time resolution. This time resolution can allow a simple mix-and-freeze experiment by, for example, placing two separate ∼500 nl drops of reactant on the piezoelectric transducer, rather than a single ∼1 ml drop. All designs for the 3D-printed and CNC-milled parts of the device, as well as the design files for the printed circuit board and the Python instrument-control software, are available at https://github.com/johnrubinstein. With these designs, scientists with limited access to technical workshops and equipment can easily construct their own device by making use of services accessed over the internet.

The intention of this open-source design is that the SIO device can be easily customized and modified for specialized applications. Various components of the device may also warrant improvements. For example, a second version of the device is already planned to allow easier positioning of the piezoelectric transducer relative to the self-wicking grid by making use of an *xyz* positioning stage. Further, a simple blotting version of the device is also being constructed that will provide the advantages of commercially available grid-freezing devices such as the Vitrobot, Gatan CP3, and Leica EM GP at a fraction of the cost and with various desired customizations. The instrument described here functions by producing an aerosol from the sample. Therefore, it should not be used when working with samples where an aerosol presents a potential health hazard, such as with some viruses, toxins or prion proteins.

## Supplementary Material

EMDB reference: apoferritin, EMD-20837

Components and sources for SIO device. DOI: 10.1107/S2059798319014372/ih5001sup1.pdf

Supplementary Video S1. Illustration of the full specimen-preparation process. DOI: 10.1107/S2059798319014372/ih5001sup2.mp4

Supplementary Video S2. Video at 480 frames per second showing a grid being plunged into liquid. DOI: 10.1107/S2059798319014372/ih5001sup3.mp4
