# Supplementary material for: Shake-it-off: a simple ultrasonic cryo-EM specimen-preparation device
Source: Acta Crystallogr D Struct Biol. 2019 Nov 22;75(Pt 12):1063–70. doi: 10.1107/S2059798319014372 (PMC6889916; doi:10.1107/S2059798319014372)
Supplement: Supplementary file 1 [file d-75-01063-sup1.pdf]

STRUCTURAL
BIOLOGY

Volume 75 (2019)

Supporting information for article:

Shake-it-off: a simple ultrasonic cryo-EM specimen-preparation device

John L. Rubinstein, Hui Guo, Zev A. Ripstein, Ali Haydaroglu, Aaron Au, Christopher M. Yip, Justin M. Di Trani, Samir Benlekbir and Timothy Kwok

Components and sources for *SIO* device

Electronic components (www.digikey.com part number):

- 40 position header (S9175-ND)
- 40 channel cable (M3AAA-4006J-ND)
- 12 position header (S7074-ND)
- 6 position header (S7039-ND)
- 2 position header (SAM15241-ND)
- 2 position shunt connector (S9341-ND)
- 2 mm × 5.5 mm power jack (CP-037AH-ND)
- pulldown resistors 10 kΩ (RNCP0603FTD10K0CT-ND) [×4]
- 0 Ω resistors (RMCF0603ZT0R00CT-ND) [×6]
- 5 V DC to DC linear regulator (1951-2747-ND)
- 12 V DC to DC linear regulator (1951-2739-ND)
- MOSFET (FQP30N06L-ND) [×4]
- 2 wire terminal block (A98333-ND) [×3]
- 3 wire terminal block (A98334-ND) [×2]
- diode (497-5765-1-ND) [×2]
- 3 pin female header connector for reed switch and infrared obstacle avoidance sensor (1568-1925-ND) [×2]

Solenoids:

- 24V 30 mm travel (Kuhnke: HD8286-R-F)
- 12V 5 mm travel (Digikey: 1528-1551-ND)

Humidifier:

- Universal USB Portable Mini Donut Float Water Humidifier, eBay Seller 8.52016 (19691)

Power supply:

- Universal 24V 6A, AC 100-240V to DC with 5mm output jack, Amazon seller Topled Light

Sensors:

- Keyestudio mini reed switch module (KY-021)
- Keyestudio Infrared Obstacle Avoidance Sensor Module (KY-032)

Structure:

- Thorlabs 12 ×12” optical breadboard
- Hillman heavy duty corner brace (from Canadian Tire) [×3]
- ¼-20 × ½” Hex cap screws to connect corner braces to optical breadboard (from Canadian Tire)
- Retort boss, VWR [×4]
- Posts are ½” diameter aluminum rods tapped for 6-32 × ½” machine screws

Adhesives (from Canadian Tire):

- Armor Coat epoxy putty repair pellets (for attachment of magnets, tweezers, piezo mount to 12 V solenoid)
- Lepage heavy duty contact cement (for attachment of piezo to piezo mount).

Control computer:

- Raspberry Pi 3B+ (www.raspberrypi.org)

Custom components (designs available on (https://github.com/johnrubinstein) :

- Magnetic tweezer connector (3D printed in polycarbonate)
- piezo mount (3D printed in polycarbonate)
- support platform for small solenoid and IR obstacle avoidance sensor (3D printed in polycarbonate) [×2]
- magnetic lock-down device (to keep 24 V solenoid in the ‘down’ position after voltage is removed (3D printed in polycarbonate)
- cryogen container (CNC milled in aluminum alloy 6061; www.3dhubs.com)
- printed circuit board (www.pcbway.com).

Supplementary Video S1. Illustration of the full specimen preparation process.

Supplementary Video S2. Video at 480 frames per second showing a grid being plunged into liquid.
